# B cells Using Calcium Signaling for Specific and Rapid Detection of *Escherichia coli* O157:H7

**DOI:** 10.1038/srep10598

**Published:** 2015-06-02

**Authors:** Ling Wang, Ronghui Wang, Byung-Whi Kong, Sha Jin, Kaiming Ye, Weihuan Fang, Yanbin Li

**Affiliations:** 1 College of Biosystems Engineering and Food Science, Zhejiang University, Hangzhou 310058, China; 2 Department of Biological and Agricultural Engineering, University of Arkansas, Fayetteville, AR 72701, USA; 3 Department of Poultry Science, University of Arkansas, Fayetteville, AR 72701, USA; 4 Department of Biomedical Engineering, Binghamton University-SUNY, Binghamton, NY 13902, USA; 5 College of Animal Sciences, Zhejiang University, Hangzhou 310058, China

## Abstract

A rapid and sensitive detection technology is highly desirable for specific detection of *E. coli* O157:H7, one of the leading bacterial pathogens causing foodborne illness. In this study, we reported the rapid detection of *E. coli* O157:H7 by using calcium signaling of the B cell upon cellular membrane anchors anti-*E. coli* O157:H7 IgM. The binding of *E. coli* O157:H7 to the IgM on B cell surface activates the B cell receptor (BCR)-induced Ca^2+^ signaling pathway and results in the release of Ca^2+^ within seconds. The elevated intracellular Ca^2+^ triggers Fura-2, a fluorescent Ca^2+^ indicator, for reporting the presence of pathogens. The Fura-2 is transferred to B cells before detection. The study demonstrated that the developed B cell based biosensor was able to specifically detect *E. coli* O157:H7 at the low concentration within 10 min in pure culture samples. Finally, the B cell based biosensor was used for the detection of *E. coli* O157:H7 in ground beef samples. With its short detection time and high sensitivity at the low concentration of the target bacteria, this B cell biosensor shows promise in future application of the high throughput and rapid food detection, biosafety and environmental monitoring.

There are estimated 48 million cases of foodborne illness resulting in 3,000 deaths, and an estimated cost of 78 billion dollars per year^1^,^2^,^3^. It has been continuously a major public health and economic burden for the United States and worldwide^4^. In particular, foodborne bacteria such as *Campylobacter jejuni*, *Listeria monocytogenes*, *Salmonella enterica*, *E. coli* O157:H7 and other shiga-toxin producing *E. coli* strains (non-O157 STEC), and *Vibrio* spp. are leading causes of foodborne diseases^5^. *E. coli* O157:H7 has been identified as a major etiologic agent, which is one cause of foodborne illness and has been found to contaminate spinach, lettuce, cider, ground beef, and cantaloupe, which is also one of the top six pathogens contributing to domestically acquired foodborne illnesses resulting in hospitalization (38%)^6^. Therefore, rapid detection of microbial pathogens in food is the solution to the prevention and recognition of problems related to health and safety.

Cell-based biosensors (CBBs) present promises of equally reliable results in much shorter times^7^. The sensing elements of CBBs could be vegetative cells of bacteria^8^,^9^, eukaryotic^10^ or mammalian cells^11^,^12^,^13^,^14^,^15^. The ability of cells to recognize and respond to stimuli has made them attractive for incorporating them into biosensors. Besides neurons, cardiac cells, adenocarcinoma cell line, more and more lymphocytes have been studied in CBBs, such as B cells, mast cells, T cells, etc^16^,^17^,^18^,^19^,^20^,^21^,^22^,^23^,^24^. Among them, B cells showed the superiority of pathogen detection in speed and sensitivity due to their capability of antigen internalization through BCR (B Cell Receptors) for processing and presentation to T cells^25^. Murine hybridoma B cells (Ped-2E9) have been used for rapid detection of pathogenic *Listeria*, the toxin listeriolysin O and the enterotoxin from *Bacillus* species^13^,^25^,^26^. Rider *et al.*^11^ used the genetically engineered B lymphocyte cells for a rapid identification of pathogens and could detect *E. coli* O157:H7 as little as 500 CFU/g in 5 min or less^11^. B cells are known as the fastest pathogen identifiers (intrinsic response in <1 s)^27^,^28^, since calcium ion plays a pivotal role in the regulation of various cellular processes in eukaryotic cells. One of the primary consequences of this identification in a B cell is that a molecule binding to BCR induces a change of Ca^2+^ flux, which is a critical event in the response of a B cell to antigen stimulation^29^.

A series of fluorescent calcium indicator dyes have been developed for measurement of free intracellular calcium in eukaryotic cells and prokaryote. Fura-2 has been known as an indicator dye for measuring the concentration of free calcium ([Ca^2+^]_i_) within living cells^30^,^31^. The ratio of fluorescence emission at the two excitation wave lengths (340:380) is considered a reliable indicator of [Ca^2+^]_i_^32^,^33^. It has been widely used but not limited in immunology, cytology and neurology for interrogating ion channels^34^,^35^,^36^. and calcium signaling^37^,^38^,^39^. However, to the best of our knowledge, there is no report on utilization of Fura-2 in B cells for the bacterial pathogen detection in the food sample.

In this study, we combined the properties of B cells and Fura-2 to develop a B cell biosensor with a low detection limit of *E. coli* O157:H7 and short detection time. The innovative approach in this study is the use of a Ca^2+^-indicator, Fura-2 for detecting BCR-induced Ca^2+^ change due to an interaction between B cells and pathogens. The reaction mechanisms underlying intracellular calcium measurement using an Ca^2+^-indicator Fura-2 are discussed elsewhere^31^. It has been long known that the excitation spectrum of Fura-2 shifts rightward upon the binding of Ca^2+^. The excitation wavelength of Fura-2 shifts from 340 nm in the presence of Ca^2+^ to 380 nm in the absence of Ca^2+^. It was found that a fluorescence ratio at 340/380 is correlated to a concentration of free intracellular Ca^2+30^.This technology has a great potential to provide a practical alternative for detection of *E. coli* O157:H7 and other pathogens.

## Results

### Principles of the B cell biosensor

**Figure 1:**
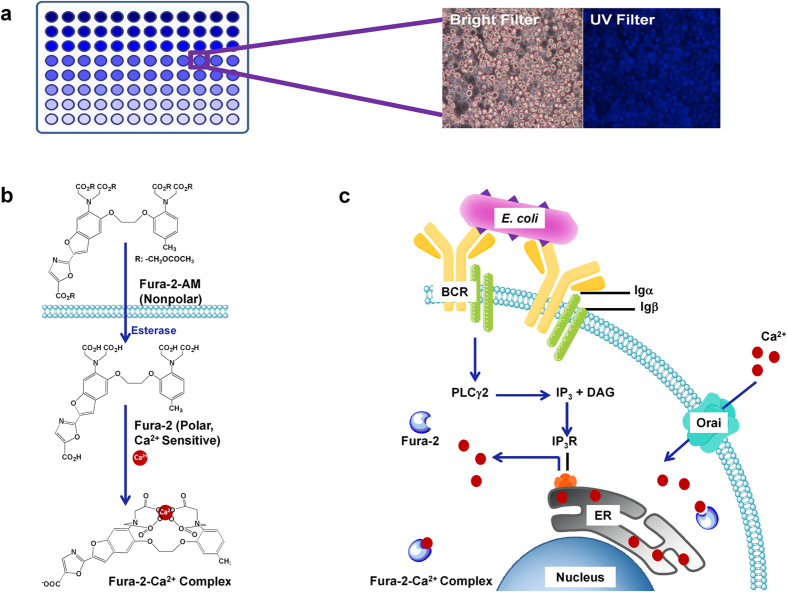
Principles of the B cell biosensor. (**a**). The response signal of the B cell biosensor was measured by a microplate reader. (**b**). Structural changes of Fura-2 by esterase activity and Ca^2+^ binding. Fura-2 AM ester is Ca^2+^ insensitive and nonpolar. Once inside the cell, esterase enzymes sequentially cleave the AM groups to leave Fura-2-free acid (Ca^2+^ sensitive, polar) trapped inside the cell, where it is able to bind Ca^2+^. (**c**). The cellular working principle of the B cell biosensor. When the target pathogen is attached to its specific receptors on the B cell surface, the cross-linking of B cell receptors (BCRs) will produce a signal, and the signaling pathways will be activated, resulting in the release of Ca^2+^ within seconds. Fura-2 exhibits a calcium dependent excitation spectral shift to report the 340/380 ratio and indicate the presence of the target pathogen.

Combining advantages of the sensitivity of Fura-2 to Ca^2+^ and the rapidness of response of B cell to antigens, a Ca^2+^-indicator based B cell biosensor was designed for *E. coli* O157:H7 detection. As shown in Fig. 1a, Fura-2 loaded B cells were placed in 96-wells, and a fluorescence ratio (340:380) was measured after the addition of analytes. Fura-2 pentaacetoxymethyl (AM) ester was used in our experiments because this form of dye is Ca^2+^ insensitive and nonpolar. Once inside the cell, esterase enzymes sequentially cleave the AM groups to leave Fura-2-free acid (Ca^2+^ sensitive, polar) trapped inside the cell and are ready for Ca^2+^ binding (Fig. 1b).

The specific B lymphocyte plays a major role in the recognition and capture of *E. coli* O157: H7. The BCR-induced Ca^2+^ flux occurs in seconds, and then B cells will exhibit increased BCR-evoked Ca^2+^ influx^29^. As shown in Fig. 1c, the B lymphocyte immune response is initiated by the binding of an antigen to the BCR^25^. The BCR is a multisubunit protein complex composed of a membrane form of immunoglobulin (Ig) that is noncovalently associated with heterodimers of Igα and Igβ. Crosslinking the BCR by the binding of bivalent or multivalent antigen leads to both transmembrane signaling and antigen internalization for presentation^40^. BCR-induced tyrosine phosphorylation of PLC-γ2 is responsible for an increase in its activity, allowing the conversion of phosphatidylinositol 4,5-bisphosphate (PIP_2_) to the second messengers inositol 1,4,5-trisphosphate (IP_3_) and diacylglycerol (DAG). IP_3_ binds IP_3_ receptor (IP_3_R), which is localized primarily on the endoplasmic reticulum (ER) and stimulates the release of calcium from intracellular stores^41^. When ER Ca^2+^ depletion occurs, Orai, a plasma membrane protein and a pore subunit of the calcium-release activated channel (CRAC), triggers CRAC activation. Thus, localized Ca^2+^ influx is activated following Ca^2+^ depletion of ER Ca^2+^ stores^29^,^42^,^43^. IP_3_ mediates a transient calcium release from intracellular stores, which leads to a sustained influx of calcium through Ca^2+^ channels in the plasma membrane, a process termed store-operated calcium entry^44^,^45^,^46^.

### Optimization of the B cell biosensor

**Figure 2:**
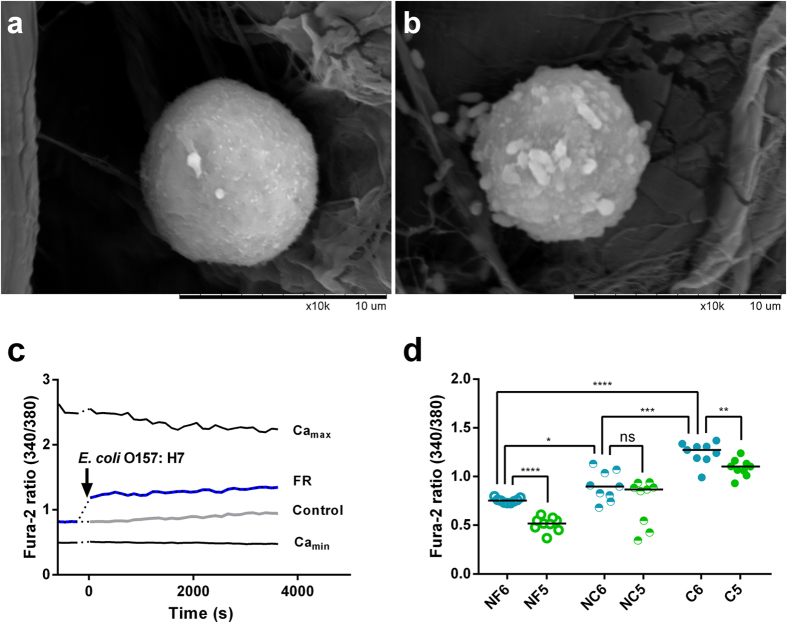
Optimization of the B cell biosensor. (**a**). SEM image of a B cell in the absence of any bacteria (×10000). (**b**). SEM image of a B cell with *E.coli* O157:H7 cells (×10000). (**c**). Real time response profile of Fura-2 340/380 ratio. (**d**). Comparison of the Fura-2 ratio for each group defined in the text. Data points represent the ratio of the stimulation by each concentration of *E. coli* O157:H7 (10^1^-10^9^ CFU/mL) in the group at the same time. Bars indicate the medians and *p*-value obtained by paired t-test. (two-tailed, ^ns^*p* > 0.05, *p < 0.05, **p < 0.01, ***p < 0.001, ****p < 0.0001, n = 9) NF6: B cells (no Fura-2, Ca^2+^ and Mg^2+^ HBSS), 10^6^ cells/mL. NF5: B cells (no Fura-2, Ca^2+^ and Mg^2+^ HBSS), 10^5^ cells/mL. NC6: B cells (Fura-2, Ca^2+^ and Mg^2+^-free HBSS), 10^6^ cells/mL. NC5: B cells (Fura-2, Ca^2+^ and Mg^2+^-free HBSS), 10^5^ cells/mL. C6: B cells (Fura-2, Ca^2+^ and Mg^2+^ HBSS), 10^6^ cells/mL. C5: B cells (Fura-2, Ca^2+^ and Mg^2+^ HBSS), 10^5^ cells/mL.

The experimental conditions such as B cell concentration, measurement time and existence of Ca^2+^ were interrogated to optimize the sensor. MARC 29F8 with membrane has monoclonal antibodies (MAbs) specific to the LPS of *E. coli* O157:H7^47^. Upon arriving, the B cells were tested for the presence of antibodies through ELISA (Enzyme-linked immunosorbent assay). Whole cell ELISA analysis procedure is given in the Supplementary Information. ELISA results (Supplementary Fig. S1) confirmed the activity of antibodies on the membrane of selected B cells and the antibodies still have activity even after 12 passages of B cells. In this study, the early-passage numbers (passage numbers between 5 and 12) of B cells were used for B cell biosensor development. The binding of *E. coli* O157:H7 to the B cells was further confirmed through scanning electron microscopy (SEM). Representative SEM micrographic images are shown in Fig. 2b.

Fura-2 ratios (*FR*) versus time were monitored using a microplate reader within 1 hour. After 10^3 ^CFU/mL of *E. coli* O157:H7 was added, the time point started as 0 min. As shown in Fig. 2c, a clearly increase in *FR* value was observed for *E. coli* O157:H7, when compared to the control (HBSS without target bacteria), which confirmed the sensing principle. In order to check the validation, further experiments were conducted to obtain the ratio of Ca^2+^-free (*Ca*_*min*_) and Ca^2+^-bound (*Ca*_*max*_) conditions. Results showed that the ratios of *FR* and *Control* were between the ratios of *Ca*_*min*_ and *Ca*_*max*_, indicating that the obtained result was valid.

The gradual increase of Fura-2 ratio was observed in both *FR* and *Control* (Fig. 2c) during the detection time from 0 to 60 min. This might be due to the leaking out of Fura-2 from the cells, which should be subtracted before the calibration is performed^32^.

To investigate the variation of the biosensor whether there was the presence of Ca^2+^ in the solution, intracellular Ca^2+^ levels were measured with and without Ca^2+^ in the solution using different concentrations of B cells with adding different concentrations of *E. coli* O157:H7 (10^1^-10^9^ CFU/mL). Paired *t*-test was conducted, as shown in Fig. 2d. In group NF (no Fura-2), B cells were not loaded with Fura-2, which was set as a blank control. The ratio obtained in NF6 (no Fura-2, 10^6^ cells/mL) was significantly higher than that obtained in NF5 (no Fura-2, 10^5^ cells/mL) (*p* < 0.0001). In group NC (no Ca^2+^), B cells were loaded with Fura-2 in the Ca^2+^ and Mg^2+^-free solution. There was no significant difference (*p* > 0.05) between NF5 (no Fura-2, 10^5^ cells/mL) and NF6. In group C (with Ca^2+^), B cells were loaded with Fura-2 in the Ca^2+^ and Mg^2+^ solution. The ratio obtained in C6 (with Ca^2+^, 10^6^ cells/mL) was much higher than that obtained in C5 (with Ca^2+^, 10^5^ cells/mL) (*p* = 0.0025). Since higher Fura-2 ratios were observed when the 10^6^ cells/mL of B cells was tested in different groups, 10^6^ cells/mL of B cells were used to carry out the comparison among the groups. The Fura-2 ratios obtained from NF6 were much lower than that obtained from NC6 (no Ca^2+^, 10^6^ cells/mL) (*p* = 0.0324) and C6 (with Ca^2+^, 10^6^ cells/mL) (*p* < 0.0001). A pronounced decrease in Ca^2+^ response (*p* = 0.0006) was observed in NC6 when the detection was carried out in the Ca^2+^ and Mg^2+^-free solution as compared to C6, where the Ca^2+^ and Mg^2+^ were present in the solution.

### Detection of *E. coli* O157:H7 in pure culture

**Figure 3:**
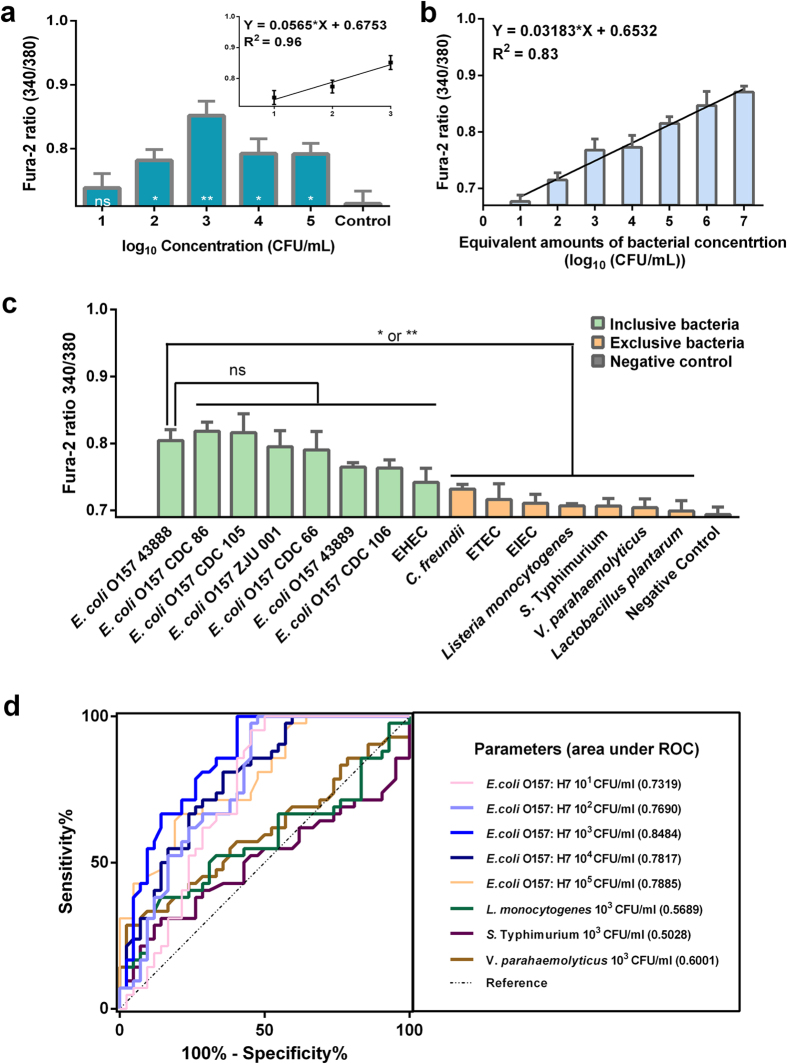
Detection of *E. coli* O157:H7 in pure culture. (**a**). Plot of the biosensor’s response, Fura-2 ratios, to different concentrations of *E. coli* O157:H7 in pure culture. Insert: Linear calibration curve obtained for *E. coli* O157:H7 in the range of concentrations from 10^1^ to 10^3^ CFU/mL. All data shown is mean±s.e.m. and *p*-value obtained by unpaired t-test. (two-tailed, ^ns^*p* > 0.05, *p < 0.05, **p < 0.01, n = 4). (**b**). Plot of the biosensor’s response, Fura-2 ratios, to different concentrations of LPS extracted from *E. coli* O157:H7 which is equal to bacterial concentrations from 10^1^ to 10^7^ CFU/mL. All data shown is mean±s.e.m. and *p*-value obtained by unpaired t-test. (two-tailed, ^ns^*p* > 0.05, *p < 0.05, **p < 0.01, n = 3). (**c**). Inclusivity tests and exclusivity tests to compare the B cell biosensor’s response to target and non-target bacteria species at 10^3^ CFU/mL. All data shown is mean±s.e.m. and *p*-value obtained by unpaired t-test. (two-tailed, ^ns^*p* > 0.05, *p < 0.05, **p < 0.01, n = 3). (**d**). ROC curves for all sensitivity sets and specificity sets with three replicates in bacteria pure culture conducted continuously for 30 min.

The developed B cell biosensor was evaluated for the detection of *E. coli* O157:H7 ranging from 10^1^ to 10^5^ CFU/mL in pure culture. The control measurement was carried out using HBSS to replace *E. coli* O157:H7. Comparing to the control, the means of the ratio were significantly different when the concentration of *E. coli* O157:H7 varies from 10^2^-10^5^ CFU/mL. As shown in Fig. 3a the ratio was found to increase linearly with the increase of the number of bacteria presented in the solution from 10^1^ to 10^3^ CFU/mL. A calibration curve was fitted by linear regression, yielding Y=0.0565X + 0.6753 (R^2^  = 0.96). When equivalent amounts of extracted LPS were used to stimulate the B cell (10^1^-10^7^ CFU/mL), the linear relation between the amount of LPS and the ratio was observed (Fig. 3b). A calibration curve was fitted by linear regression, yielding Y=0.03183X + 0.6532 (R^2^ = 0.83).

Selective detection of *E. coli* O157:H7 is necessary for use of the biosensor in food safety. Therefore, the abilities of the B cell biosensor to detect target organisms and non-target organisms were determined by the inclusivity test and the exclusivity test, respectively. Eight strains of *E. coli* comprising 2 different serogroups (O157 and non-O157) were tested, and seven of the strains, except for EHEC (non-O157), were sensitive for the detection in inclusivity tests. Exclusivity tests using 7 strains from 6 distinct different genera showed high specificity (Fig. 3c). *p*-values of the results of inclusivity and exclusivity tests are shown in Supplementary Table S5.

*Listeria monocytogenes*, *S.* Typhimurium and *V. parahaemolyticus* were chosen as representative bacteria in exclusivity tests, because this B cell was constructed to produce monoclonal antibodies (MAbs) specific for the LPS of *E. coli* O157 and group N *Salmonella* for use as highly specific diagnostic reagents. Bio X cell (West Lebanon, NH, USA) has developed anti-mouse IgM isotype control (Catalog number: BE0087) using B cell MARC 29F8. Mabs 29F8 was core antigen specific and also was determined to react with the LPS core antigen on the basis of its reactivity with the 12- to 16-kDa bands of the aqueous-phase LPS^47^. *L. monocytogenes* and *V. parahaemolyticus* were used as the representatives of Gram-positive and Gram-negative bacteria respectively. As to *S.* Typhimurium, it was used to test the specificity of this biosensor to O-antigen. The polysaccharides of group N *Salmonella* such as *S. godesberg* belong to chemotype VI (basal sugars with additional galactosamine and fucose). *S.* Typhimurium belongs to group D (polysaccharides chemotype XVI: basal sugars with additional mannose, rhamnose and tyvelose)^48^.

The accuracy of this sensor was evaluated quantitatively by Receiver Operating Characteristic (ROC) curve (Fig. 3d). The ratios of Fura-2 in the presence of *E. coli* O157:H7 ATCC 43888 at different concentrations respectively were detected continuously for 30 min, and each concentration was repeated three times. The collected data was used to obtain the ROC curve. The accuracy of the detection as represented by the area under ROC (AUR) curves was determined to be 0.7319, 0.7690, 0.8484, 0.7817 and 0.7885, respectively, for the concentrations of *E. coli* O157:H7 from 10^1^, 10^2^, 10^3^,10^4^ to 10^5^ CFU/mL. All values of AUR obtained from different concentrations of *E. coli* O157:H7 were greater than 0.7 in a range of 0.7319 to 0.8484, indicating that the B cell biosensor was accurate in the detection range. It is observed that AUR value dropped to 0.7817 and 0.7885 when the concentration of *E. coli* O157:H7 increased to 10^4^ CFU/mL and 10^5^ CFU/mL, respectively. When 10^3^ CFU/mL *L. monocytogenes*, *S.* Typhimurium, and *V. parahaemolyticus* were tested, the AUR values dropped to 0.5689, 0.5028 and 0.6001, respectively.

### Detection of *E. coli* O157:H7 in ground beef

**Figure 4:**
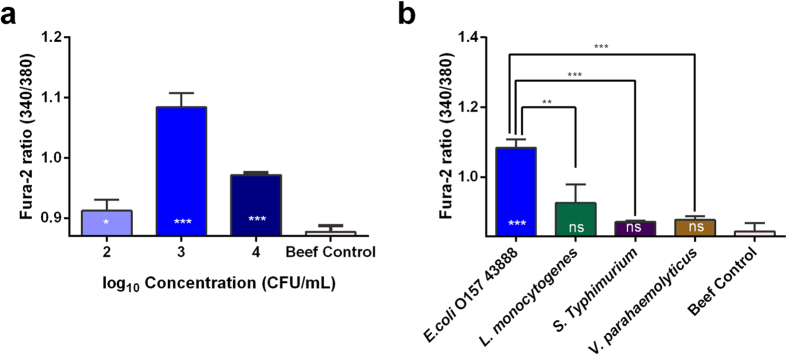
Detection of *E. coli* O157:H7 in ground beef. (**a**). Plot of the biosensor’s response, Fura-2 ratio, to different concentrations of *E. coli* O157: H7 in ground beef. All data shown is mean±s.e.m. and *p*-value obtained by unpaired t-test. (two-tailed, *p < 0.05, ***p < 0.001, n = 3). (**b**). Biosensor’s response to different pathogens with the same concentration of 10^3^ CFU/mL in ground beef. All data shown is mean±s.e.m. and *p*-value obtained by unpaired t-test. (two-tailed, ^ns^*p* > 0.05, **p < 0.01, ***p < 0.001, n =3)

The B cell biosensor was further evaluated with bacteria inoculated ground beef extract samples. Based on the experiments performed using pure culture, we chose ground beef extract that were inoculated with 10^2^ CFU/mL, 10^3^ CFU/mL and 10^4^ CFU/mL *E. coli* O157:H7 for the test. The B cells’ response to the ground beef extract inoculated with *E. coli* O157:H7 ATCC 43888 exhibited a similar response to the pure culture. A control measurement was carried out in the absence of *E. coli* O157:H7. As shown in Fig. 4a, significant differences, as compared to a control, were observed when the *E. coli* O157:H7 concentrations varied from 10^2^-10^4^ CFU/mL (*p* = 0.0420, 0.0002 and 0.0001 respectively).

*L. monocytogenes*, *S.* Typhimurium and *V. parahaemolyticus* were used in ground beef samples as non-target bacteria to evaluate the specificity of the biosensor. As shown in Fig. 4b, when the same concentration (10^3^ CFU/mL) of different pathogen was detected, the ratio of the sample with *E. coli* O157:H7 was extremely significant compared to the control (*p* = 0.0002), but ratios of Fura-2 with non-target bacteria weren’t found the significant differences compared to the control (*p* > 0.05) in the ground beef extract samples. The very significant difference between the detection of *E. coli* O157:H7 and *L. monocytogenes* was found (*p* = 0.0095), and the extremely significant differences between the detection of *E. coli* O157:H7 and *S.* Typhimurium and *V. parahaemolyticus* were found (*p* = 0.0001 and 0.0002 respectively).

## Discussion

The proposed Ca^2+^ indicator based B cell biosensor for detecting *E. coli* O157:H7 with better combined detection time and sensitivity was conceived, tested and validated in this study. BCR-induced Ca^2+^ flux can be triggered in seconds, and the Ca influx will happen instantly^29^, leading to Fura-2 (a Ca^2+^ sensitive divalent metal ion chelator) complexing with Ca^2+33^, all of them happened in a very short time. In the optimization of the B cell biosensor, our data suggested that the non-subtracted ratios obtained from the earlier time would be more accurately correlated to *E. coli* O157:H7. To determine the optimum time for the detection, the ratio of *FR* obtained from the different time was subtracted by the ratio of *Control* at the same time. It was found that after four to six min the response signal became stable. Thus, the ratio at 10^th^ min was used as a detection ratio in the subsequent experiments. ROC curves drawn using the data collected from continuous 30 min in the bacterial pure culture (Fig. 3d) showed the same results as that at the 10^th^ min.

From Fig. 2d, it is very clear that the B cells with the higher concentration combined with relatively the same concentration of *E. coli* O157:H7 had higher ratio produced by autofluorescence signal of bacteria, and the ratio produced by the autofluorescence of B cells without loading Fura-2 combined with *E. coli* O157:H7 had significant differences from the ratio produced by the B cells loaded with Fura-2 when the detection solution was with or without Ca^2+^ and Mg^2+^. Considering the previous result obtained from the comparison between group NC5 and NC6, these data divulged that a proportion of the total BCR induced Ca^2+^ flux observed is due to extracellular Ca^2+^ influx. The previous research on CD20 induced cytosolic Ca^2+^ flux also indicated a proportion of the total Ca^2+^ flux observed is due to extracellular Ca^2+^ influx in B cells^49^. The aforementioned results showed that the autofluorescence signal from the organism was negligible in comparison with the Fura-2 response signal. The higher response signal could be obtained from the higher concentrations of the B cells because more Ca^2+^ influx from the extracellular would increase the ratio, which would increase the biosensor sensitivity. Therefore, 10^6^ cells/mL B cells loaded with Fura-2 in the solution with Ca^2+^ were used in the later tests.

In the detection of *E. coli* O157:H7 in pure culture, the B cell biosensor provided an increasing signal response which is linear in relation to the logarithm of *E. coli* O157:H7 concentration (10^1^-10^3^ CFU/mL). The ratio began to drop when the concentration of *E. coli* O157:H7 was 10^4^ CFU/mL and 10^5^ CFU/mL, probably because the high number of *E. coli* cells triggered the B cell expansion and apoptosis^50^, subsequently BCR-induced Ca^2+^ signaling was interfered. To verify it, the equivalent amounts of extracted LPS from *E. coli* O157:H7 were detected by this B cell biosensor. As shown in Fig. 3a, the ratio increased linearly with an increase of the equivalent amounts of LPS (10^1^-10^7^ CFU/mL). It is consistent with a recent study showing the ability of B cells to present antigen-derived peptides to T cells was also dependent on the amount of antigen tethered on the membrane^51^.

In the selective detection of *E. coli* O157:H7 in pure culture, our results showed that nonspecific binding did not lead to significant increase in the ratio with respect to the control in this B cell biosensor. Our results also showed that the MAbs on the surface of B cells are specific to O-antigen and the LPS of Gram-negative bacteria, which might have caused less response to EHEC (non-O157) and Gram-positive bacteria such as *L. monocytogenes* and *Lactobacillus plantarum*, respectively. In Fig. 3c, the statistical analysis of *p*-values (based on t-test) shows significant differences between inclusive bacteria and exclusive bacteria (Supplementary Table S5), however, it is recognized that the signal of all *E. coli* O157:H7 in the inclusivity test may need to be further amplified to ensure the true positive or true negative results for real food sample testing.

Based on ROC curve in Fig. 3d, it is quite evident that the B cell biosensor has a good specificity and its detection is reproducible and the ratio obtained from the 10^th^ min could represent the results obtained from 10 min to 30 min during the detection. Since there was no significant difference as compared to the control when *E. coli* O157:H7 concentration was 10^1^ CFU/mL, the detection range of the biosensor for *E. coli* O157:H7 was 10^2^-10^5^ CFU/mL.

For the detection of bacteria in the pure culture, this B cell biosensor had a detection limit of 5.9×10^2^ CFU/mL. An additional feature of this biosensor is its rapid analysis time of 10 min. It is shorter than most biosensing methods which have the detection limits in the range of 10^2^ to 10^4^ CFU/mL, such as surface plasmon resonance (SPR)^52^, enzyme-linked immunosorbent assay (ELISA)^53^, fiber optic^54^ and quartz crystal microbalance (QCM)^55^. *E. coli* O157:H7 bacteria as low as 7 CFU were detected on the screen-printed carbon electrode (SPCE) sensor in 70 min^56^, and 10 CFU/mL were detected by the long-range surface plasmon-enhanced fluorescence spectroscopy (LRSP-FS) in 40 min^57^. There are also some biosensors with a low detection limit and a short detection time, such as 67 CFU/mL were detected by a lateral-flow immunobiosensor based on electrospun nanofibers and conductive magnetic nanoparticles (MNPs) in 8 min^58^, 500 CFU/g in lettuce were also detected by CANARY (cellular analysis and notification of antigen risks and yields) sensor in less than 5 min^11^. In this study, since only 30 μL test sample was used in each well for detection, the B cell biosensor was actually able to detect 18 cells of *E. coli* O157:H7. If some concentration procedures were applied in sample preparation, the detection limit of the B cell biosensor could be further improved to the level of several cells per mL. This biosensor can be easily operated in sample handling and preparation for multiplex tests, which has the potential for rapid, high throughput detection of foodborne pathogens.

For the detection of bacteria in ground beef, this B cell biosensor had a detection limit of 8.6×10^2^ CFU/mL (26 CFU in 30 μL test sample) responding to *E. coli* O157:H7 in the ground beef extract sample. According to the statistical analysis, the specificity significance of target and non-target bacteria detected in the pure culture and the ground beef are slightly different. It may be due to food particulates and high levels of background microbiota from beef extract affectted the sensitivity of this B cell biosensor.

The advantages of the rapid response to the antigen of B cell and the sensitivity to Ca^2+^ of Fura-2 were combined to develop this Ca^2+^ -indicator based B cell biosensor which successfully detected *E. coli* O157:H7 at a low detection limit and in short time in both pure culture and ground beef. The main hurdle that remains is that the B cells themselves are not comparably ‘stable’ to other biosensing elements such as antibodies or nucleic acid probes. The signal response varies from batch to batch, and is influenced mainly by the cell’s viability, cell concentration and the exposing time after loading Fura-2. To overcome this hurdle, freshly prepared B cells are preferred, and the controls need to be employed for each batch test.

In summary, B cell MARC 29f8 loaded with Ca^2+^ -indicator Fura-2 was successfully developed as a convenient and sensitive biosensor. By using *E. coli* O157:H7 as a model target pathogen, the target bacteria were detected by measuring the intracellular Ca^2+^ concentration caused by BCR induced Ca^2+^ flux. This B cell based biosensor combined the properties of the sensitivity to Ca^2+^ of Fura-2 and the rapid response to the antibody of B cells.

The results showed that the responses of the developed B cell biosensor were sensitive and specific with a detection limit as low as 10^2^ CFU/mL in both pure culture and ground beef within 10 min. The combination of the fluorescently loaded B cells specifically against *E. coli* O157:H7 and a microplate reader will pave the way for designing B cell based biosensors with features of easy operation, low-cost and high throughput for food detection of pathogens in food safety, biosafety, clinical diagnostics and environmental monitoring.

## Methods

### Reagents

Dulbecco’s modified Eagle’s medium (DMEM), Dulbecco’s modified Eagle’s medium without phenol red (DMEM without PR), heat-inactivated fetal bovine serum (FBS), Hank’s balanced salt solution (HBSS), Ca^2+^ and Mg^2+^-free HBSS, MEM non-essential amino acids (MEM NAA), 0.4% Trypan Blue Solution, Fura-2/AM and 0.5 M EDTA were purchased from Life Technologies (Carlsbad, CA, USA). All bacteria culture mediums were purchased from BD (Sparks, MD, USA). Goat antimouse IgM-HRP (horseradish peroxidase), Lipopolysaccharide (LPS) extraction kit and silver staining kit were obtained from AbD (AbD Serotec, USA), Intron Biotechnology (Gyeonggi-do, Korea) and Sangon Biotech (Shanghai, China), respectively. All other reagents were obtained from Sigma (St. Louis, MO, USA). All solutions and buffers used in this study were prepared in sterile de-ionized (DI) water from Millipore Direct-8 system (Billerica, MA, USA).

### B cell lines and culture conditions

The B cell line MARC 29F8 was obtained from ATCC (American Type Culture Collection, Manassas, VA; ATCC number CRL-2508). The organism of this B cell line is mouse, cell type is hybridoma B lymphocyte, and isotype is IgM. It was routinely cultured in DMEM supplemented with 4 mM L-glutamine adjusted to contain 1.5 g/L sodium bicarbonate, 4.5 g/L glucose, 1 vol% MEM NAA and 10% heat-inactivated FBS. The cells were cultured at 37 °C in a humid atmosphere containing 7% CO_2._ Liquid-nitrogen-stored MARC 29F8 cells (passage numbers between 5 and 10) were suspended at a ratio of 1:10 in the complete medium and grown for 72 h. MARC 29F8 cells were passaged again in DMEM with or without phenol red supplemented with 10% heat-inactivated FBS in T-25 and T-75 flasks (Falcon, Oxnard, USA) until they reached to a logarithmic phase of growth (usually 72 h) after which they were used for experiments. The cell count was performed using a TC10 Automated Cell Counter (Bio-Rad, Hercules, CA, USA) according to the manufacturer’s instructions.

### Bacterial strains and culture conditions

Bacterial strains used in this study were obtained from the American Type Culture collection (ATCC), China Center of Industrial Culture Collection (CICC), China National Center for Medical Culture Collections (CMCC), the Chinese Zhejiang Center for Disease Control and Prevention (Zhejiang Province CDC) and Zhejiang University (ZJU). The detailed informations are shown in Supplementary Table S1 and Table S2.

*Lactobacillus plantarum* and other strains were grown on MRS broth and BHI broth at 37 °C, respectively. *Listeria monocytogenes* and other strains were cultured 48 h and 24 h, respectively. The concentration of bacteria in this study was determined in triplicate by enumeration on Trypticase Soy Agar (TSA). The detailed information is described in the Supplementary Information.

**Scanning electron microscopy (SEM) imaging** SEM samples were prepared (see the Supplementary Information) and imaged using a Hitachi TM1000 scanning electron microscope (SEM) (Hitachi, Japan).

### Preparation of a B cell biosensor

The B cell biosensor was constructed under sterile conditions by preparing B cells loaded with Ca^2+^ -indicator Fura-2, with a slight modification to the measurement of [Ca^2+^]_i_ in whole cell suspensions using Fura-2^33^. In brief, for fluorescent Ca^2+^ flux experiment, undifferentiated MARC 29F8 cells were harvested in DMEM without PR and incubated at 37 °C for 5 min. B cells were washed three times in HBSS after DMEM (without PR) was removed. Then the cell suspensions were incubated with 4.5 nmol of Fura-2/AM per 10^6^ cells for 30 min at 37 °C in the dark and the cells sediment were resuspended in 30 mL HBSS buffer. After incubating for an additional 15 min at room temperature in the dark to allow for the de-esterification of the Fura-2/AM, the cells were sedimented, and then split and resuspended in 30 mL of HBSS buffer and Ca^2+^ and Mg^2+^-free HBSS buffer twice respectively.

### Detection of *E. coli* O157:H7 in pure culture

Briefly, a portion of 30 μl Fura-2 loaded MARC 29F8 B cells were added into each well of a 96-well plate, and then 30 μl analytes were loaded into the wells. To assess dynamic ranges for the microplate reader, maximum and minimum fluorescence values (at 340 and 380 nm excitation wavelengths) were determined in separate experiments in which Fura-2 loaded MARC 29F8 cells were incubated with 0.1% Triton X-100 and 4.5 mM ethylenediamine-tetraacetic acid (EDTA), respectively. Fluorescence was evoked by 340- and 380-nm excitation wavelengths (F340 and F380) and collected at 510 nm using Synergy H1 Hybrid Multi-Mode Microplate reader (BioTek, Winooski, VT, USA). Data was collected every 120 s in the plate reader. Data from all measurements were presented as 340/380 fluorescence ratios directly representative of changes in intracellular Ca^2+30^. *E. coli* O157:H7 ATCC 43888 was serially diluted in HBSS from 6.2×10^1^ to 6.2×10^9^ and from 5.9×10^1^ to 5.9×10^5^ in the optimization test and the accuracy test, respectively.

Equivalent amounts of extracted LPS from *E. coli* O157:H7 ATCC 43888 were used to verify the linear relation between the bacterial concentration and the response of the B cell biosensor. The detailed information is described in the Supplementary Information.

Totally, 8 strains and 7 strains at the bacterial concentration of 10^3^ CFU/mL were used for inclusivity tests and exclusivity tests, respectively (Supplementary Table S1 and Table S2). *E. coli* O157:H7 ATCC 43888 and HBSS without any bacteria were added into Fura-2 loaded B cells as the positive control and negative control, respectively. The sensor response to different concentrations of bacteria was calculated as the mean value of ratios measured in three independent tests.

### Detection of *E. coli* O157:H7 in ground beef

To validate the developed B cell biosensor, experiments were conducted using ground beef extract. The ground beef were purchased from a local grocery store, and contaminated with three different concentrations of *E. coli* O157:H7 to mimic bacterial contamination. Twenty-five grams of ground beef was mixed with 225 ml of HBSS in Filtra bags (Labplas Inc., Quebec, Canada) and stomached with Stomacher 400 (Seward, Norfolk, UK) for 1 min. Then the washing solution was collected. One milliliter of *E. coli* O157:H7 cells at dilutions of 10^3^, 10^4^, and 10^5^ CFU/mL was added to 9 ml of washing solution of the ground beef sample, to obtain the ground beef samples contaminated with *E. coli* O157:H7 at 10^2^, 10^3^, and 10^4^ CFU/mL. Then the same procedure for detection of *E. coli* O157:H7 in HBSS was followed using the prepared ground beef extract samples. The Fura-2 loaded B cells in the ground beef extract sample were used as a control for the test.

### Statistical analysis

Data represents mean±s.e.m. (standard error of the means) of three experiments. The data were analyzed by GraphPad Prism software (GraphPad, San Diego, CA). Paired t-test was used in section **Optimization of the B cell biosensor** to analyze the differences between two groups which were both added with nine different concentrations of *E. coli* O157: H7 respectively. Unpaired t-test was used in the rest of experiments to analyze the differences between two groups which were added with the same concentration of bacterial pathogens. *p* < 0.05 were considered significant.

## Additional Information

**How to cite this article**: Wang, L. *et al*. B cells Using Calcium Signaling for Specific and Rapid Detection of *Escherichia coli* O157:H7. *Sci. Rep.* **5**, 10598; doi: 10.1038/srep10598 (2015).

## Supplementary Material

Supplementary Information
