# Supplementary material for: B cells Using Calcium Signaling for Specific and Rapid Detection of Escherichia coli O157:H7
Source: Sci Rep. 2015 Jun 2;5:10598. doi: 10.1038/srep10598 (PMC4451841; doi:10.1038/srep10598)
Supplement: Supplementary Information [file srep10598-s1.pdf]

# B cells Using Calcium Signaling for Specific and Rapid Detection of *Escherichia coli* O157:H7

Ling Wang[1,2], Ronghui Wang[2], Byung-Whi Kong[3], Sha Jin[4], Kaiming Ye[4], Weihuan Fang[5], Yanbin Li[1,2*]

[1]College of Biosystems Engineering and Food Science,
Zhejiang University, Hangzhou 310058, China

[2]Department of Biological and Agricultural Engineering,
University of Arkansas, Fayetteville, AR 72701, USA

[3]Department of Poultry Science,
University of Arkansas, Fayetteville, AR 72701, USA

[4]Department of Biomedical Engineering,
University of Arkansas, Fayetteville, AR 72701, USA

[5]College of Animal Sciences,
Zhejiang University, Hangzhou 310058, China

*Corresponding author:
Yanbin Li, Professor
Tel: +86 57188982536
Fax: +86 57188982530
E-mail address: yanbinli@zju.edu.cn (Y. Li)

## Supplementary Information

**Bacterial strains and concentration determination**

**Table S1 *S*trains used in the inclusivity test.**

**Table S2 Strains used in the exclusivity test.**

**Scanning electron microscopy (SEM) imaging**

**Whole cell ELISA (Enzyme-linked immunosorbent assay)**

**Figure S1 Absorbance of whole-cell ELISA of the B cells.**

**Detection of *E. coli* O 157:H7 in pure culture**

**Detection of LPS**

**Figure S2 Comparison between the extracted LPS and the control LPS.**

**Detection of *E. coli* O 157:H7 in solid ground beef**

**Figure S3 Detection of *E. coli* O 157:H7 inoculated in solid ground beef samples**

**Complementarily statistical data**

**Table S3 Statistical data used in Fig. 2d (paired t-test, n = 9).**

**Table S4 Mean and SEM values of Fura-2 ratios measured in the biosensor's response to different concentrations of *E. coli* O157:H7 in pure culture (unpaired t-test, n = 4).**

**Table S5 Statistic data for all bacteria tested and shown in Fig. 3c (unpaired t-test, n = 3)**

**Table S6 Statistical data used in Fig. 3d (unpaired t-test, n = 42).**

**Table S7 Statistical data used in Fig. 4a (unpaired t-test, n = 3).**

**Table S8 Statistical data used in Fig. 4b (unpaired t-test, n=3).**

**Bacterial strains and concentration determination**

The concentration of bacteria in this study was determined in triplicate by enumeration on TSA agar following incubation at 37℃ for 24 h and 48 h (*L. monocytogenes*). One milliliter of the frozen bacterium stationary phase culture was freshly thawed and inoculated into 9 mL of BHI broth or MRS broth and incubated. The fresh culture of bacteria were washed three times by centrifugation at 8000 rpm for 5 min and the pellets were resuspended in HBSS. A portion (100 μL) of the resuspension was spread plated onto TSA agar in triplicate, incubated at 37°C for 24 h, resultant colonies were enumerated, and the bacterial culture concentration was back calculated.

**Table S1 *S*trains used in the inclusivity test.**

| Organism | Strain / Source | Isolation |
|---|---|---|
| Enterohemorrhagic *Escherichia. coli* (EHEC) non-O157 | CDC | Feces of patient, Zhejiang, China |
| *Escherichia. coli* O157:H7 | ATCC 43888 | Human feces |
| *Escherichia. coli* O157:H7 | ATCC 43889 | Feces of patient, North Carolina, USA |
| *Escherichia. coli* O157:H7 | CDC 66 | Feces of patient, Zhejiang, China |
| *Escherichia. coli* O157:H7 | CDC 86 | Feces of patient, Zhejiang, China |
| *Escherichia. coli* O157:H7 | CDC 105 | Feces of patient, Zhejiang, China |
| *Escherichia. coli* O157:H7 | CDC 106 | Feces of patient, Zhejiang, China |
| *Escherichia. coli* O157:H7 | ZJU 001 | Feces of patient, Zhejiang, China |

**Table S2. Strains used in the exclusivity test.**

| Organism | Strain / Source | Isolation |
|---|---|---|
| *Citrobacter freundii* | CMCC 48001 | Patient, China |
| Enteroinvasive *Escherichia. coli* (EIEC) | CDC | Feces of patient, Zhejiang, China |
| Enterotoxigenic *Escherichia. coli* (ETEC) | CDC | Feces of patient, Zhejiang, China |
| *Lactobacillus plantarum* | CICC 21790 | Pickled vegetable |
| *Listeria monocytogenes* | ATCC 19115 | Patient |
| *Salmonella* Typhimurium | ATCC 14028 | Tissue, chicken |
| *Vibrio parahaemolyticus* | ATCC 33847 | Patient, Maryland, USA |

**Scanning electron microscopy (SEM) imaging**

B cells were mixed with *E. coli* for 10 min and then processed using the following procedures for SEM. The sample was fixed with 2.5% paraformaldehyde for 16 h and then post fixed with 1% osmium tetroxide for 2 h. The samples were then dehydrated using 30, 50, 70, 80, 90, and 100% graded ethanol for 15 min each, a mixture of ethanol and iso-amyl acetate (v:v=1:1) for 30 min, and then pure isoamyl acetate for 1 h. The sample was dehydrated in a Hitachi Model HCP-2 critical point drier with liquid $CO_2$. The dehydrated sample was coated with gold-palladium in a Hitachi model E-1010 ion sputter for 5 min and observed using a Hitachi model TM-1000 SEM.

**Whole cell ELISA (Enzyme-linked immunosorbent assay)**

Since the specificity of the B cells were already tested [1], only the activity and the stability of the B cell (passage 9 and passage 12) were tested by whole cell ELISA in this study. In brief, 200 μL $10^8$ cells/mL cultured B cells were added into 96-well plates. The

cells were washed twice by centrifugation at 1400 rpm for 5 min and the pellets were resuspended in 200 μL FCS (fetal calf serum ) buffer (1% BSA in PBS). 50 μL goat antimouse IgM-HRP (horseradish peroxidase ) (AbD, Serotec) with the dilution in PBS 1:900 was added into wells and incubated on ice for 1 hour. After the cells were washed three times by FCS buffer as described previously, 100 μL of TMB (3,3′,5,5′-tetramethylbenzidine, Sigma) substrate buffer was added into the wells. The plate was incubated at 37°C for 50 min and the absorbance was measured at 450 nm in a microplate reader. For the control sample, PBS was used to replace B cells.

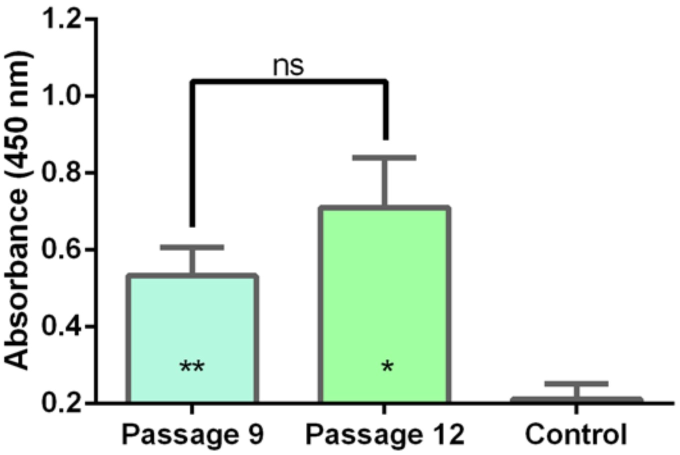


**Figure S1 Absorbance of whole-cell ELISA of the B cells.** Results are presented according to different passages (passage 6 and passage 12, cell concentrations are $10^8$ cells/mL). All data shown is mean±s.e.m. and *p*-value obtained by unpaired t-test. (two-tailed, $^{ns}p > 0.05$, $*p < 0.05$, $**p < 0.01$, n = 3)

The absorbance of different passages of B cells (passage 9 and passage 12) was not significantly different ($p > 0.05$, ns) while the absorbance of them was highly significant comparing to the control ($p = 0.0026$ and 0.0193 respectively). These results demonstrated that the B cells have the IgM activity on the surface and also indicated that

IgM on the surface was stable from passage 9 to passage 12. Thus the B cells with the passages 6-12 were used in this study.

**Detection of LPS**

LPS were extracted with the LPS extraction kit (Intron Biotechnology, Gyeonggi-do, Korea) according to the manufacturer's instructions and samples were analyzed by SDS-PAGE as previously described[2]. Silver staining was performed by using the Silver staining kit (Sangon, Shanghai, China). Briefly, 5 mL of 24 h-grown culture was used for the extraction, and the final LPS was dissolved in 50 μL sterile deionized water. Afterwards, 10 mL aliquots of the extracted LPS and the control LPS purchased from Sigma (1 mg/mL) were loaded onto SDS-PAGE gels, respectively. In the experiment of the LPS detection, 10 mL extracted LPS was equal to the amount of 1 mL $10^8$ CFU/mL *E. coli* O157:H7, and then was serially diluted in HBSS.

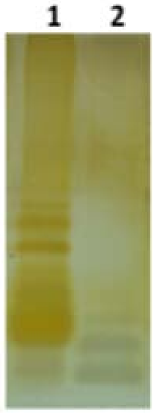


**Figure S2 Comparison between the extracted LPS and the control LPS.**

Lane 1: the extracted LPS of *E. coli* O157:H7.

Lane 2: the control LPS of *E. coli* purchased from Sigma.

**Detection of *E. coli* O 157:H7 in solid ground beef**

Five grams grounded beef samples were weighed aseptically and placed into sterile stomacher bags. The bags containing grounded beef were inoculated with 0.1 mL *E. coli* O157:H7 ATCC 43888 to obtain a concentration of approx. 3 $log_{10}$ CFU/g for the samples, and 0.1 mL HBSS for the blank control, respectively. Thereafter, the bags were compressed into a thin layer (approx. 1 to 2 mm thick) by pressing against a flat surface, excluding most of the air, and then heat sealed. The sealed bags were blended for 2 min and then were place at the room temperature for 16 h. After 16 h enrichment incubation, 45 mL HBSS was added into each sample bags, and then 30 μL extracts were used as the analytes to perform the B cell biosensor test. The result showed the ratio obtained from inoculated beef sample was significantly higher than the ratio obtained in the beef control ($p < 0.001$).

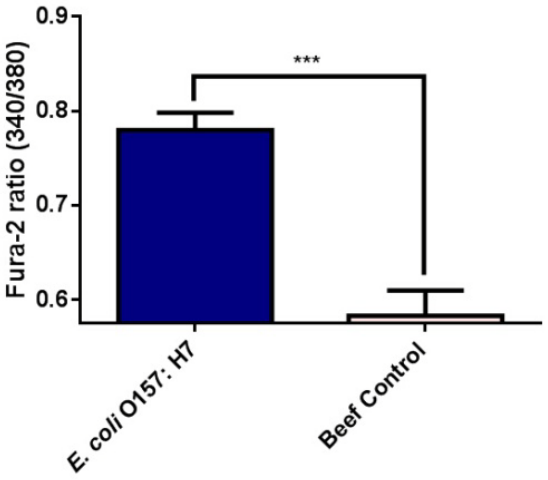


**Figure S3 Detection of *E. coli* O 157:H7 inoculated in solid ground beef samples**
Plot of the biosensor's response, Fura-2 ratio, to $10^3$ CFU/mL *E. coli* O157: H7 inoculated in ground beef. All data shown is mean ± s.e.m. and *p*-value obtained by unpaired t-test. (two-tailed, ***$p < 0.001$, n = 3)

**Complementarily statistical data**

**Table S3 Statistical data used in Fig. 2d (paired t-test, n = 9).**

| | Group vs. Group | | | | | |
|---|---|---|---|---|---|---|
| | **NF6 vs. NF5** | **NC6 vs. NC5** | **C6 vs. C5** | **NF6 vs. NC6** | **NF6 vs. C6** | **NC6 vs. C6** |
| ***p* value (two-tailed)** | <0.0001 | 0.1582 | 0.0025 | 0.0324 | <0.0001 | 0.0006 |
| **Mean of differences** | 0.2420 | 0.1577 | 0.1531 | 0.1462 | 0.4888 | 0.3426 |
| **SD* of differences** | 0.05663 | 0.3039 | 0.1061 | 0.1698 | 0.1004 | 0.1884 |
| **SEM* of differences** | 0.01888 | 0.1013 | 0.03536 | 0.05660 | 0.03347 | 0.06279 |

***SD**: Standard deviation.

**SEM**: Standard error of the mean.

**Table S4 Mean and SEM values of Fura-2 ratios measured in the biosensor's response to different concentrations of *E. coli* O157:H7 in pure culture (unpaired t-test, n = 4).**

| **Group** | **1*** | **2*** | **3*** | **4*** | **5*** | **C*** |
|---|---|---|---|---|---|---|
| **Mean** | 0.7388 | 0.7818 | 0.8518 | 0.7923 | 0.7915 | 0.7140 |
| **SEM*** | 0.02246 | 0.01696 | 0.02246 | 0.02325 | 0.01696 | 0.01971 |
| **Group vs. Group C** | | **1 vs. C** | **2 vs. C** | **3 vs. C** | **4 vs. C** | **5 vs. C** |
| ***p*-values (two-tailed)** | | 0.4393 | 0.0404 | 0.0037 | 0.0425 | 0.0246 |

***1:** *E. coli* O157:H7, $10^{1}$ CFU/mL, pure culture.

**2:** *E. coli* O157:H7, $10^{2}$ CFU/mL, pure culture.

**3:** *E. coli* O157:H7, $10^{3}$ CFU/mL, pure culture.

**4:** *E. coli* O157:H7, $10^4$ CFU/mL, pure culture.

**5:** *E. coli* O157:H7, $10^5$ CFU/mL, pure culture.

**C:** Negative control.

**SEM**: Standard error of the mean.

**Table S5 Statistic data for all bacteria tested and shown in Fig. 3c (unpaired t-test, n = 3)**

| Strains | Mean | SEM* | *p*-values (two-tailed) | |
|---|---|---|---|---|
| | | | Strains vs. Negative Control | Strains vs. Positive Control (*E. coli* O157 43888) |
| *E. coli* O157:H7 43888 | 0.8043 | 0.01636 | 0.0051 (**) | |
| *E. coli* O157:H7 CDC 86 | 0.8184 | 0.01335 | 0.0021 (**) | |
| *E. coli* O157:H7 CDC 105 | 0.8161 | 0.02840 | 0.0172 (*) | |
| *E. coli* O157:H7 ZJU 001 | 0.7951 | 0.02385 | 0.0185 (*) | |
| *E. coli* O157:H7 CDC 66 | 0.7904 | 0.02745 | 0.0312 (*) | |
| *E. coli* O157:H7 43889 | 0.7644 | 0.006736 | 0.0058 (**) | |
| *E. coli* O157:H7 CDC 106 | 0.7632 | 0.01222 | 0.0140 (*) | |
| EHEC | 0.7417 | 0.02118 | 0.1165 (ns) | |
| *C. freundii* | 0.7317 | 0.007248 | | 0.0064 (**) |
| ETEC | 0.7163 | 0.02342 | | 0.0369 (*) |
| EIEC | 0.7105 | 0.01336 | | 0.0065 (**) |
| *Listeria monocytogenes* | 0.7067 | 0.003231 | | 0.0043 (**) |
| *S.* Typhimurium | 0.7063 | 0.01135 | | 0.0037 (**) |
| *V. parahaemolyticus* | 0.7040 | 0.01320 | | 0.0048 (**) |
| *Lactobacillus plantarum* | 0.6993 | 0.01514 | | 0.0055 (**) |

($^{ns}p > 0.05$, $*p < 0.05$, $**p < 0.01$)

***SEM**: Standard error of the mean

**Table S6 Statistical data used in Fig. 3d (unpaired t-test, n = 42)**

| **Group vs. Group C*** | **AUR** | **SD*** | ***p*-values (two-tailed)** |
|---|---|---|---|
| **1* vs. C** | 0.7319 | 0.05750 | 0.0002559 |
| **2* vs. C** | 0.7690 | 0.05247 | <0.0001 |
| **3* vs. C** | 0.8484 | 0.04224 | <0.0001 |
| **4* vs. C** | 0.7817 | 0.04974 | <0.0001 |
| **5* vs. C** | 0.7885 | 0.04809 | <0.0001 |
| **L* vs. C** | 0.5689 | 0.06396 | 0.2771 |
| **S* vs. C** | 0.5028 | 0.06510 | 0.9643 |
| **V* vs. C** | 0.6001 | 0.06265 | 0.1144 |

***1:** *E. coli* O157:H7, $10^1$ CFU/mL, pure culture.

**2:** *E. coli* O157:H7, $10^2$ CFU/mL, pure culture.

**3:** *E. coli* O157:H7, $10^3$ CFU/mL, pure culture.

**4:** *E. coli* O157:H7, $10^4$ CFU/mL, pure culture.

**5:** *E. coli* O157:H7, $10^5$ CFU/mL, pure culture.

**L:** *L. monocytogenes*, $10^3$ CFU/mL, pure culture.

**S:** *S.* Typhimurium, $10^3$ CFU/mL, pure culture.

**V:** *V. parahaemolyticus*, $10^3$ CFU/mL, pure culture.

**C:** Negative Control

**SD:** Standard deviation

**Table S7 Statistical data used in Fig. 4a (unpaired t-test, n = 3).**

| **Group** | **2*** | **3*** | **4*** | **C*** |
|---|---|---|---|---|
| **Mean** | 0.9123 | 1.084 | 0.9717 | 0.8773 |
| **SEM*** | 0.01037 | 0.01358 | 0.002404 | 0.005783 |
| **Group vs. C** | **2** vs. **C** | **3** vs. **C** | **4** vs. **C** | |
| ***p*-values (two-tailed)** | 0.0420 | 0.0002 | 0.0001 | |

***2:** *E. coli* O157:H7, $10^2$ CFU/mL, ground beef.

**3:** *E. coli* O157:H7, $10^3$ CFU/mL, ground beef.

**4:** *E. coli* O157:H7, $10^4$ CFU/mL, ground beef.

**C**: Negative Control, ground beef.

**SEM**: Standard error of the mean

**Table S8 Statistical data used in Fig. 4b (unpaired t-test, n = 3).**

| **Group** | **E*** | **L*** | **S*** | **V*** | **C*** |
|---|---|---|---|---|---|
| **Mean** | 1.084 | 0.9255 | 0.8710 | 0.8773 | 0.8433 |
| **SEM*** | 0.01358 | 0.03109 | 0.002309 | 0.005783 | 0.01426 |
| **Group vs. Control** | **E vs. C** | **L vs. C** | **S vs. C** | **V vs. C** | |
| ***p*-values (two-tailed)** | 0.0003 | 0.0742 | 0.1280 | 0.0917 | |
| **Group E vs. Group** | **E** vs. **L** | **E** vs. **S** | **E** vs. **V** | | |
| ***p*-values (two-tailed)** | 0.0095 | 0.0001 | 0.0002 | | |

***E:** *E. coli* O157:H7, ground beef.

**L:** *L. monocytogenes*, ground beef.

**S:** *S.* Typhimurium, ground beef.

**V:** *V. parahaemolyticus*, ground beef.

**C:** Negative control, ground beef.

**SEM**: Standard error of the mean.

## References

S1. Westerman, R.B., He, Y., Keen, J.E., Littledike, E.T. & Kwang, J. Production and characterization of monoclonal antibodies specific for the lipopolysaccharide of *Escherichia coli* O157. *Journal of clinical microbiology* **35**, 679-684 (1997).

S2. Lerner, A.*, et al.* The Azospirillum brasilense Sp7 noeJ and noeL genes are involved in extracellular polysaccharide biosynthesis. *Microbiology* **155**, 4058-4068 (2009).
